# Two-Sample Mendelian Randomization Analysis of Associations Between Periodontal Disease and Risk of Cancer

**DOI:** 10.1093/jncics/pkab037

**Published:** 2021-04-19

**Authors:** Laura Corlin, Mengyuan Ruan, Konstantinos K Tsilidis, Emmanouil Bouras, Yau-Hua Yu, Rachael Stolzenberg-Solomon, Alison P Klein, Harvey A Risch, Christopher I Amos, Lori C Sakoda, Pavel Vodička, Pai K Rish, James Beck, Elizabeth A Platz, Dominique S Michaud

**Affiliations:** 1 Department of Public Health and Community Medicine, Tufts University School of Medicine, Boston, MA, USA; 2 Department of Civil and Environmental Engineering, Tufts University School of Engineering, Medford, MA, USA; 3 Department of Epidemiology and Biostatistics, School of Public Health, Imperial College London, London, UK; 4 Department of Hygiene and Epidemiology, University of Ioannina School of Medicine, Ioannina, Greece; 5 Department of Periodontology, Tufts University School of Dental Medicine, Boston, MA, USA; 6 National Cancer Institute, Rockville, MD, USA; 7 Department of Oncology, Johns Hopkins University School of Medicine, Baltimore, MD, USA; 8 The Sidney Kimmel Comprehensive Cancer Center at Johns Hopkins, Baltimore, MD, USA; 9 Department of Chronic Disease Epidemiology, Yale School of Public Health, New Haven, CT, USA; 10 Department of Medicine, Baylor College of Medicine, Waco, TX, USA; 11 Division of Research, Kaiser Permanente Northern California, Oakland, CA, USA; 12 Department of the Molecular Biology of Cancer, Institute of Experimental Medicine, Academy of Sciences of the Czech Republic, Prague, Czech Republic; 13 Laboratory Medicine and Pathology, The Colon Cancer Family Registry at Mayo Clinic, Rochester, MN, USA; 14 Department of Dental Ecology, University of North Carolina, Chapel Hill, NC, USA; 15 Department of Epidemiology, Johns Hopkins Bloomberg School of Public Health, Baltimore, MD, USA for CCFR, CORECT, GECCO, ILCCO, PanScan, and PanC4

## Abstract

**Background:**

Observational studies indicate that periodontal disease may increase the risk of colorectal, lung, and pancreatic cancers. Using a 2-sample Mendelian randomization (MR) analysis, we assessed whether a genetic predisposition index for periodontal disease was associated with colorectal, lung, or pancreatic cancer risks.

**Methods:**

Our primary instrument included single nucleotide polymorphisms with strong genome-wide association study evidence for associations with chronic, aggressive, and/or severe periodontal disease (rs729876, rs1537415, rs2738058, rs12461706, rs16870060, rs2521634, rs3826782, and rs7762544). We used summary-level genetic data for colorectal cancer (n = 58 131 cases; Genetics and Epidemiology of Colorectal Cancer Consortium, Colon Cancer Family Registry, and Colorectal Transdisciplinary Study), lung cancer (n = 18 082 cases; International Lung Cancer Consortium), and pancreatic cancer (n = 9254 cases; Pancreatic Cancer Consortia). Four MR approaches were employed for this analysis: random-effects inverse‐variance weighted (primary analyses), Mendelian Randomization-Pleiotropy RESidual Sum and Outlier, simple median, and weighted median. We conducted secondary analyses to determine if associations varied by cancer subtype (colorectal cancer location, lung cancer histology), sex (colorectal and pancreatic cancers), or smoking history (lung and pancreatic cancer). All statistical tests were 2-sided.

**Results:**

The genetic predisposition index for chronic or aggressive periodontitis was statistically significantly associated with a 3% increased risk of colorectal cancer (per unit increase in genetic index of periodontal disease; *P* = .03), 3% increased risk of colon cancer (*P* = .02), 4% increased risk of proximal colon cancer (*P* = .01), and 3% increased risk of colorectal cancer among females (*P* = .04); however, it was not statistically significantly associated with the risk of lung cancer or pancreatic cancer, overall or within most subgroups.

**Conclusions:**

Genetic predisposition to periodontitis may be associated with colorectal cancer risk. Further research should determine whether increased periodontitis prevention and increased cancer surveillance of patients with periodontitis is warranted.

Colorectal cancer, lung cancer (including tracheal and bronchus cancers), and pancreatic cancer together account for >2.9 million deaths per year globally (1). Colorectal and lung cancer have the 2 highest number of incident cases globally (1.7 million and 2.0 million annual cases, respectively). Most histologic subtypes of lung cancer and pancreatic cancer have poor prognoses because, even in the United States, at least 50% of cases are not diagnosed until the cancer is at a less curative, advanced stage (2). Primary prevention efforts are thus critical, and observational studies indicate that modifiable risk factors (eg, periodontal disease) are implicated in the pathogenesis of colorectal, lung, and pancreatic cancers (3-5). Although well-conducted meta-analyses of observational studies have strengthened the evidence for positive associations between periodontal disease and lung, colorectal, and pancreatic cancers (6-8), additional evidence for causal associations could be observed in randomized trials or in observational studies that employ methods that emulate randomization (eg, Mendelian randomization [MR]).

Two-sample MR is an approach that uses summary association estimates (often from genome-wide association studies [GWAS]) to develop a genetic instrument index for the exposure and then applies the index to assess the association with the outcome in a different sample of the same underlying source population (9,10). The instrument must be associated with the exposure, associated with the outcome only through paths that include the exposure, and independent of exposure-outcome confounders (11). Two-sample MR has advantages over 1-sample MR: weak instrument bias tends to drive association estimates towards the null in 2-sample MR rather than in the direction of the observational associations as in 1-sample MR, and robust instruments from larger GWAS can be used in 2-sample MR investigations such that more precise and accurate estimates may be obtained (12-14).

Previous 2-sample MR studies used genetic instruments for periodontal disease (15-17) and other studies used 2-sample MR to assess risk factors for each of colorectal, lung, and pancreatic cancer (18-20); however, to our knowledge, no MR study has assessed the association between periodontal disease and cancer risk. Given the need to rigorously assess putative causal relationships among modifiable factors (eg, oral health) and cancer risk to support health promotion, our primary objective was to assess whether genetic predisposition to having chronic or aggressive periodontal disease was associated with colorectal, lung, or pancreatic cancers using a 2-sample MR analysis. Our secondary objectives were to assess whether these associations varied by cancer subtype (location in the large bowel for colorectal cancer and histology for lung cancer), sex (for colorectal and pancreatic cancers), or smoking history (for lung and pancreatic cancers) and were robust against potential violations of MR assumptions.

## Methods

### Genetic Instrument for Periodontal Disease

We determined 2 genetic instruments for periodontal disease (defined in the Supplementary Methods, available online) based on a systematic evaluation of the strength of the GWAS evidence for associations between individual single nucleotide polymorphisms (SNPs) and chronic, aggressive, and/or severe periodontal disease (Supplementary Table 1, available online) (21-28). There were 8 SNPs in our primary instrument, of which 5 had very strong evidence for an association with periodontal disease (rs729876, rs1537415, rs2738058, rs12461706, rs16870060) and 3 had strong evidence (rs2521634, rs3826782, rs7762544) (21-25). We considered the evidence very strong if the association with periodontitis met the genome-wide statistical significance threshold of *P* < 5 × 10^−8^ in a pooled analysis of multiple cohorts (focusing on populations of European descent to match the population demographics of our outcome data). We considered the evidence strong for SNPs that were positively associated with chronic periodontitis in 1 cohort (*P* < 5 × 10^−6^), nominally positively associated (*P* < .05) with severe chronic periodontitis in an independent replication cohort, and positively associated (*P* < 5 × 10^−6^) with chronic periodontitis in a meta-analysis of over 5000 European American individuals. Our secondary instrument included the 8 SNPs in the primary instrument and 6 additional SNPs (rs1122900, rs2064712, rs2070901, rs4970469, rs9982623, rs9984417) with moderate evidence for an association with periodontitis (statistically significant with a threshold of *P* < 5 × 10^−6^ in a pooled analysis of multiple cohorts but not associated with periodontitis in any single cohort with a threshold of *P* < 5 × 10^−6^) (21,22). We assessed whether any of the 14 SNPs were potentially pleiotropic, in mutual linkage disequilibrium, accounted for population stratification, or were problematic to harmonize. The Supplementary Methods (available online) contain additional details about our SNP selection process. We include both the primary and secondary instruments so that the reader can assess the sensitivity of the results to our SNP selection process and to balance the advantages of having more SNPs in the instrument with having SNPs with the strongest evidence for an association.

### Summary-Level Data for Lung, Colorectal, and Pancreatic Cancer

We used summary-level genetic data for colorectal cancer (overall, by location in the large bowel, and by sex) from 125 478 participants (including 58 131 colorectal cancer and advanced adenoma cases) in the Genetics and Epidemiology of Colorectal Cancer Consortium (GECCO; 13 studies), Colon Cancer Family Registry (CCFR), and Colorectal Transdisciplinary study (CORECT) (29-31); lung cancer (overall, by histologic type, and by smoker status [current or noncurrent]) from 31 862 participants (including 18 082 cases) in the International Lung Cancer Consortium (ILCCO; 26 studies included) (32,33); pancreatic cancer (overall, by sex, smoker status, data source [pancreatic cancer consortium], and study design) for 13 823 participants (including 5090 cases) in PanScan I and II (12 cohort and 8 case-control studies) and PanScan III (15 cohorts, 2 case series, and 1 case-control study) (30,34); and pancreatic cancer (overall, by sex, and by smoker status) in 7956 participants in the Pancreatic Cancer Case-Control Consortium (PanC4; including 4164 cases for a total of 9254 pancreatic cancer cases) (35-37). Choices for secondary analyses (eg, not assessing colorectal cancer associations by smoker status) were due to data availability. All cancer data came from individuals of European ancestry. All studies participating in each consortium obtained informed consent from participants and approval from the relevant ethical review boards. None of the study samples that contributed genetic data for lung, colorectal, or pancreatic cancer overlapped with study samples that contributed data for the periodontitis GWAS. Genotyping and imputation methods for each consortium have been described previously and are summarized in the Supplementary Methods (available online) (29,37-45).

Because we used only deidentified data, we did not need institutional review board approval for our analysis.

### Statistical Analyses

In our primary analysis, we estimated the association between each genetic predisposition index for having chronic or aggressive periodontal disease and colorectal, lung, and pancreatic cancer risks using the random-effects inverse-variance weighted (IVW) method. We also considered 3 other MR estimation methods (Mendelian Randomization-Pleiotropy RESidual Sum and Outlier (MR-PRESSO), simple median, and weighted median) with different assumptions to evaluate the robustness of the IVW findings. A description of the strengths and weaknesses of each MR approach is provided in the Supplementary Methods (available online). We quantitatively assessed violations of the “NO Measurement Error” assumption using the $I_{\mathrm{GX}}^{2}$ statistic (where values between 0.9 and 1 suggest that violations are negligible and that the uncertainty in the SNP exposure associations are substantially smaller than the underlying heterogeneity in these associations) (46,47). We assessed horizontal pleiotropy using the *I^2^* statistic (where values >50% indicate potential horizontal pleiotropy) (48). All statistical analyses were performed in R (version 3.6.2) with packages MendelianRandomization (version 0.4.2) and *MRPRESSO* (version 1.0). We constructed plots showing the genetic associations of the SNPs with periodontitis (natural log values of the odds ratios shown in Supplementary Table 1, available online) vs the genetic associations of the SNPs with each cancer (natural log values of the odds ratios provided by each consortium) using *ggplot2* (slope represents the beta values for the MR models). Default parameters were used for each analysis. All statistical tests were 2-sided. Our cut point for statistical significance was .05.

## Results

**Figure 1.**
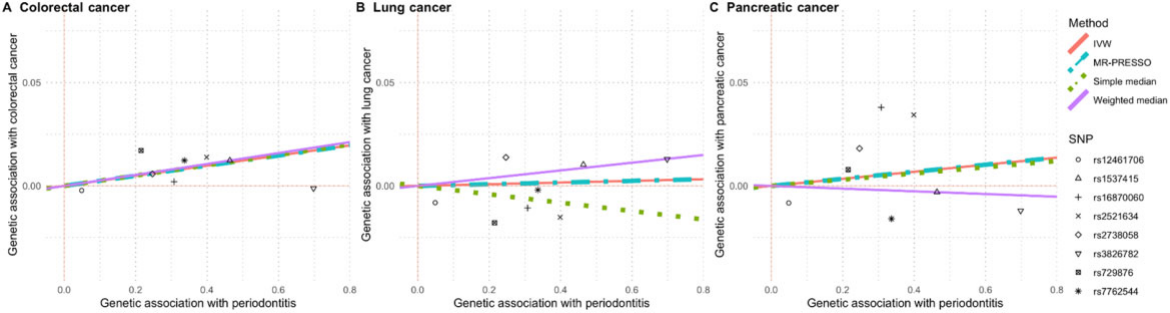
Scatterplots comparing the strength of the Single Nucleotide Polymorphism (SNP)–exposure (periodontitis) and SNP–outcome (cancer risk) associations. The lines indicate the estimated effect sizes by 4 Mendelian Randomization (MR) methods (Inverse-Variance Weighted [IVW], MR-PRESSO, simple median, and weighted median).

Using the 8 SNPs with the strongest evidence for a genetic predisposition to having chronic or aggressive periodontal disease, we observed a statistically significant association with the risk of colorectal cancer (3% increase per unit increase in genetic index of periodontal disease; *P* = .03) but not with the risk of lung (0.4% increase; *P* = .83) or pancreatic cancers (2% increase; *P* = .51; Table 1; Figure 1). In secondary analyses, including an additional 6 SNPs with moderately strong evidence for an association with periodontitis attenuated the effect estimates for the association with colorectal cancer but did not substantially change the effect estimates for either lung or pancreatic cancers. For the primary and secondary analyses, $I_{\mathrm{GX}}^{2}$ values were 0.947 and 0.926, respectively, indicating that the effect estimates were unlikely to be substantially biased towards the null due to violations of the no measurement error assumption. There was no indication of horizontal pleiotropy for any of the primary analyses (*I^2^* of 0% for each colorectal, lung, and pancreatic cancers) and limited evidence of horizontal pleiotropy for the secondary analyses (*I^2^* of 60% for colorectal, 12% for lung, and 0% for pancreatic cancer). Separate sensitivity analyses removing each of rs1537415 (palindromic allele), rs3826782 (low effect allele frequency and potentially influential), rs12461706 (palindromic allele), rs1537415 and rs12461706 (palindromic alleles in the primary instrument), and rs9984417 (palindromic allele) did not substantively change any of these results (Supplementary Table 2, available online).

**Table 1.** Effect estimates for the association between genetic predisposition to having chronic or aggressive periodontitis and the risk of colorectal, lung, and pancreatic cancer by genetic instrument and MR approach

Cancer outcome	N_cases_/N_controls_	Instrument^a^	IVW^b^	MR-PRESSO	Simple median	Weighted median
			β (*P*)	β (*P*)	β (*P*)	β (*P*)
Colorectal	58131/67347	Primary	0.025 (.03)	0.025 (.01)	0.025 (.12)	0.027 (.06)
		Secondary	0.006 (.70)	0.016 (.11)	0.002 (.88)	0.025 (.05)
Colon	31083/67347	Primary	0.031 (.02)	0.031 (<.001)	0.030 (.10)	0.030 (.08)
		Secondary	0.010 (.47)	0.010 (.48)	0.017 (.34)	0.027 (.07)
Rectal	15775/67347	Primary	0.002 (.93)	0.002 (.94)	−0.015 (.55)	0.011 (.64)
		Secondary	−0.014 (.47)	−0.014 (.49)	−0.043 (.09)	0.004 (.87)
Lung	18082/13780	Primary	0.004 (.83)	0.004 (.76)	−0.020 (.48)	0.019 (.45)
		Secondary	−0.006 (.75)	−0.006 (.75)	−0.011 (.66)	0.017 (.45)
Pancreatic	9254/12525	Primary	0.017 (.51)	0.017 (.41)	0.015 (.70)	−0.007 (.85)
		Secondary	0.021 (.34)	0.021 (.26)	0.055 (.11)	0.011 (.71)

a The primary analysis included 8 SNPs (rs729876, rs1537415, rs2738058, rs12461706, rs16870060, rs2521634, rs3826782, and rs7762544). The secondary analysis included 6 additional SNPs (rs1122900, rs2064712, rs2070901, rs4970469, rs9982623, and rs9984417). IVW = inverse-variance weighted; MR-PRESSO = Mendelian Randomization Pleiotropy RESidual Sum and Outlier; SNP = single nucleotide polymorphism.

b The primary Mendelian randomization method (ie, statistical test) was inverse-variance weighted (IVW) MR. We used MR-PRESSO, simple median, and weighted median as secondary analyses. Betas indicate the effect estimate for the association between a 1-unit increase in genetic predisposition to having chronic or aggressive periodontitis and the natural log risk for each cancer outcome. All statistical tests were 2-sided.

**Figure 2.**
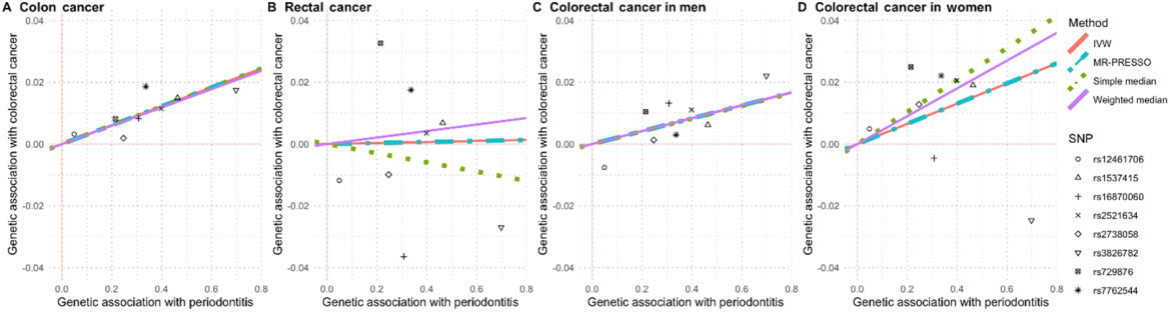
Scatterplots comparing the strength of the Single Nucleotide Polymorphism (SNP)–exposure (periodontitis) and SNP–colorectal cancer associations. The lines indicate the estimated effect sizes by 4 Mendelian Randomization (MR) methods (Inverse-Variance Weighted [IVW], MR-PRESSO, simple median, and weighted median).

In addition to the analyses for each cancer overall, we assessed associations between genetic predisposition to having chronic or aggressive periodontitis and risk of colorectal cancer stratified by location in the large bowel (colon, rectal, distal, and proximal) and sex (Figure 2; Supplementary Figure 1, available online). We observed that each unit increase in the genetic predisposition index for chronic or aggressive periodontitis was associated with a 3% increased risk in colon cancer (*P* = .02), a 4% increased risk of proximal colon cancer (*P* = .01), and a 3% increased risk of colorectal cancer among females (*P* = .04; Figure 2). Each of these associations was observed with the IVW MR method and at least 1 alternative MR approach (Supplementary Table 3, available online). Additionally, whereas the primary analyses using the MR approach did not suggest statistically significant associations with rectal cancer (β = 0.002, *P* = .93), distal cancer (β = 0.023, *P* = .19), or colorectal cancer in men (β = 0.021, *P* = .17), with the MR-PRESSO method, a 1-unit increase in the genetic predisposition index for chronic or aggressive periodontitis was associated with a 2% increased risk of distal colorectal cancer (*P* = .03) and a 2% increased risk of colorectal cancer in men (*P* = .01). In secondary analyses including the 6 additional SNPs with moderate evidence for an association with periodontitis, none of the associations assessed with any of the MR methods remained statistically significant (Supplementary Table 3, available online).

We also investigated associations between genetic predisposition to having chronic or aggressive periodontitis and risk of lung cancer stratified by histologic type (adenocarcinoma, squamous cell, or small cell), smoker status (current or not current), and the combination of histologic type and smoker status (Table 2). We did not observe statistically significant associations for any of these analyses using the primary genetic instrument or with any of the MR methods (Supplementary Table 4, available online). However, using the secondary genetic instrument, a 1-unit increase in genetic predisposition index for chronic or aggressive periodontitis was associated with a 34% decreased risk of small cell lung cancer among nonsmokers (*P* = .02; Supplementary Table 4, available online). Notably, this secondary analysis included a very small number of cases (n = 64) and may thus simply represent statistical noise (especially because the association with the primary instrument was not statistically significant with a β of −0.383 and a *P* value of .06).

**Table 2.** Effect estimates for the association between genetic predisposition to having chronic or aggressive periodontitis^a^ and the risk of lung cancer by histologic subtype and smoker status

Lung cancer	Overall		Smokers		Nonsmokers	
	(N_controls_ = 13 780)		(n_controls_ = 9084)^b^		(n_controls_ = 4415)	
	N_cases_	β^c^ (*P*)	n_cases_	β (*P*)	n_cases_	β (*P*)
Overall	18 082	0.004 (.83)	15 984	0.002 (.93)	1800	−0.021 (.65)
Adenocarcinoma	6730	0.025 (.31)	5639	0.029 (.32)	975	0.012 (.84)
Squamous cell	4429	−0.010 (.74)	4209	−0.019 (.54)	158	−0.040 (.77)
Small cell	1853	−0.035 (.40)	1761	−0.023 (.59)	64	−0.383 (.06)

^a^The inverse-variance weighted Mendelian randomization analysis included 8 SNPs (rs729876, rs1537415, rs2738058, rs12461706, rs16870060, rs2521634, rs3826782, and rs7762544). SNP = single nucleotide polymorphism.

^b^Controls were shared across lung cancer histologic subtypes.

^c^Betas indicate the effect estimate for the association between a 1-unit increase in genetic predisposition to having chronic or aggressive periodontitis and the natural log risk for each lung cancer outcome. All statistical tests were 2-sided.

For pancreatic cancer, we observed no statistically significant associations when we stratified by sex or smoker status (Table 3). In addition, results were similar by study design (cohort or case control; Supplementary Table 5, available online) and by dataset (ie, PanScan I and II, PanScan III, and Pancreatic Cancer Case-Control Consortium; data not shown). In general, the main analysis results were not substantively different than the results from the secondary analyses including the 6 additional SNPs (Supplementary Tables 5 and 6, available online); the only exception was for a separate analysis of the PanScan cohort studies where positive associations were observed with pancreatic cancer using both the IVW (15% increased risk, *P* = .02) and MR-PRESSO methods (15% increased risk, *P* = .01; Supplementary Table 5, available online).

**Table 3.** Effect estimates for the association between genetic predisposition to having chronic or aggressive periodontitis^a^ and the risk of pancreatic cancer by sex and smoker status using PanScan and PanC4 data

Pancreatic cancer	No. of cases/controls	IVW
		β (*P*)^b^
Overall	9254/12 525	0.017 (.51)
Female	4243/4734	0.036 (.40)
Male	5011/7791	0.003 (.94)
Current smoker	1517/1724	0.053 (.44)
Former smoker	3286/4982	−0.008 (.88)
Never smoker	3314/5199	0.020 (.63)

a The inverse-variance weighted (IVW) Mendelian randomization analysis included 8 SNPs (rs729876, rs1537415, rs2738058, rs12461706, rs16870060, rs2521634, rs3826782, and rs7762544). PanC4 = Pancreatic Cancer Case-Control Consortium; SNP = single nucleotide polymorphism.

b Betas indicate the effect estimate for the association between a 1-unit increase in genetic predisposition to having chronic or aggressive periodontitis and the natural log risk for pancreatic cancer. All statistical tests were 2-sided.

## Discussion

Using data from several large cancer consortia and a genetic instrument index for predisposition to having chronic or aggressive periodontal disease developed through a rigorous systematic selection process, we conducted the first, to our knowledge, 2-sample MR assessment of periodontitis in relation to the risks of developing colorectal, lung, and pancreatic cancer. We observed evidence that a genetic predisposition to having chronic or aggressive periodontitis is associated with colorectal cancer (overall, and in a subanalysis only including women), colon cancer, and proximal colon cancer. Conversely, our 2-sample MR results were not consistent with the hypothesis that genetically predicted periodontal disease is linked to lung cancer or pancreatic cancer risk.

Our observation of an MR association between genetic predisposition to periodontitis and increased colorectal cancer risk is supported by several observational studies (4,49,50), though not all (51). Additionally, our observation that the relationship between genetic predisposition to periodontal disease and colorectal cancer risk varies by sex is supported by null associations in an all-male cohort (52), positive associations in 1 all-female cohort (53), and suggestive positive associations in another small all-female cohort (n = 19 participants with colorectal cancer) (54); however, a large cohort study with clinical measurements for periodontal disease reported similar positive associations in men and women (55). More studies will need to examine the role of sex in the association between periodontal disease and colorectal cancer.

Plausible causal mechanisms linking periodontal disease to colorectal cancer incidence may involve inflammatory processes or oral microbiome shifts (dysbiosis) that migrate to extra oral sites (56,57). For example, the gram-negative *Fusobacteria* is among the quantitatively dominant microorganisms in dental plaque (58); it interacts with inflammatory processes associated with periodontal disease, and it has been identified in colorectal cancer tissue (58-60). Notably, the proportion of colorectal cancer cases with high *Fusobacteria* varies by location (generally observed more in proximal vs distal cases) (61-63). Furthermore, microbiota organization (eg, presence of a bacterial biofilm) is particularly associated with proximal colon cancer compared with distal colon cancer (64). These observations, along with studies indicating that multiple environmental factors and mutation profiles have differential associations by cancer location in the large bowel (65-68), support our finding that genetic predisposition to periodontal disease may be more likely to influence proximal colon cancer risk than distal colon cancer or rectal cancer risk.

In contrast to meta-analyses of observational studies suggesting that periodontal disease is associated with the risk of lung and pancreatic cancer (6-8,69,70), our primary analyses did not indicate that there were statistically significant associations between a genetic predisposition to periodontal disease and the risk of either lung or pancreatic cancer. Given that the effect estimates for colorectal cancer and pancreatic cancer were similar but we had over 6 times as many colorectal cancer cases, our pancreatic cancer analyses may have been underpowered. Similarly, we had over 3 times as many colorectal cancer cases as lung cancer cases. Additionally, residual confounding by smoking status could explain some of the differences between our results and those from certain observational studies (especially due to the null results in our secondary analyses including only nonsmokers) (6). The statistically significant association we observed in 1 analysis with the secondary genetic instrument using only data from PanScan cohort study participants was most likely a chance finding, or it could suggest that other pathways are involved that we failed to capture with our existing primary instrument. Finally, based on our overall results, it is possible that periodontal disease is not causally involved with lung or pancreatic cancer initiation and may instead be linked with cancer progression. This hypothesis would be supported by evidence that cancer progression is related to increased presence of certain oral bacteria common among individuals with periodontal disease (71) or is affected by oxidative stress, inflammatory, or immunological responses associated with periodontal disease (72-74). More research using markers of periodontal disease that may affect cancer progression could provide insight into this alternative scenario.

As with all MR studies, 1 limitation of our analysis is the potential for violations of the MR assumptions. For example, we could not directly test for the presence (or impact of) directional pleiotropy using MR-Egger due to the small number of genetic variants in our instrument. However, the overall consistency of our primary analyses using the IVW method and secondary analyses using different MR methods (MR-PRESSO, simple median, and weighted median—an approach that is less biased by the presence of directional pleiotropy) (75) suggests both that directional pleiotropy is unlikely to completely explain the results and that outliers were unlikely to substantially affect the results. One exception to this general trend was observed in the analysis for colorectal cancer in men where the effect estimates using the IVW and MR-PRESSO methods were identical, but the association was only statistically significant using the MR-PRESSO method. Bias could also arise due to assortative mating (ie, if parental genotypes are correlated) (76,77). There could also be concerns about the interpretability of the results (particularly with the IVW method) if potential gene–environment interactions led to violations of the assumption that the genetic instrument level modified any effect of periodontitis on cancer (78) or if the association with periodontitis did not fulfill the monotonicity assumption for other reasons (79). Additionally, the primary phenotypes associated with at least several of the SNPs used in the analysis (eg, rs2738058, rs12461706, and rs2070901) involve inflammatory pathways. Because inflammation processes are likely on a putative causal pathway between periodontal disease and cancer risk, our choice of SNPs may introduce vertical pleiotropy and potentially strengthen the genetic instrument. Finally, we may have observed statistically significant associations by chance due to the multiple comparisons made, we did not have data stratified by smoker status for colorectal data or smoking data that distinguished former vs never smokers for lung cancer, and our study results are only generalizable to individuals of European ancestry.

Strengths of our study include the large number of cancer cases included in each analysis, our MR approach that limited the potential for confounding or reverse causation, and our systematic approach to SNP selection for inclusion in our genetic instrument. Previous 2-sample MR analyses that used genetic instruments for periodontal disease (examining noncancer outcomes) included SNPs identified from single GWAS articles without clear justification for using those specific articles and SNPs (15,17). Another 2-sample MR analysis included SNPs that were not statistically significantly associated with periodontitis as well as SNPs that were statistically significantly associated with the autoimmune outcomes (rheumatoid arthritis and systemic lupus erythematosus) in GWAS (potentially introducing bias due to violations of the MR assumptions) (16,80). In contrast, we examined the strength of the evidence for an association of each SNP with periodontal disease based on objective criteria (eg, inclusion of validation and replication cohorts, definition of periodontal disease). We also quantitatively assessed our assumptions about these criteria. Finally, our inclusion of SNPs associated with aggressive (early onset) periodontitis may reflect the risk of periodontal disease only rather than possible shared risk factors of periodontal disease and cancer.

Our 2-sample MR analysis utilizing a systematically developed genetic instrument suggests that a genetic predisposition to having chronic or aggressive periodontal disease may be associated with colorectal cancer risk. Additionally, our results suggest confounding is unlikely to fully explain previous observational studies’ claims for an association between periodontal disease and colorectal cancer. Our results were not consistent with the hypothesis that a genetic predisposition to having periodontal disease is associated with lung or pancreatic cancer risk; however, we cannot entirely rule out the possibility that periodontal disease is associated with either of these cancers. Taken together, our results suggest that increased attention to preventative oral health measures and increased cancer surveillance of patients with periodontitis may be warranted. Future research is needed to further elucidate biological pathways underlying the associations between periodontitis and cancer risk.

## Funding

This work was supported the AACR-Johnson & Johnson Lung Cancer Innovation Science grant number 18–90-52-MICH. LC was supported by the National Institute of Child Health & Human Development at the National Institutes of Health (grant number K12HD092535). KKT was supported by Cancer Research UK (grant number C18281/A29019). CA was a Research Scholar supported by Cancer Prevention Research Institute of Texas grant RR170048. Funding for the cancer consortia that provided genetic data for this analysis is listed in the Supplementary Material (available online).

## Notes

**Role of the funder:** None of the funders had any role in this analysis or interpretation of the data; the writing of the manuscript; or the decision to submit this manuscript for publication.

**Disclosures:** No authors have any conflicts of interest to disclose.

**Author contributions:** Conceptualization: EAP, DSM. Data curation: MR, DSM, consortia (CCFR, CORECT, GECCO, ILCCO, PanScan, and PanC4). Validation: KKT, EB. Formal analysis: MR. Supervision: DSM. Writing—Original Draft: LC. Writing—Review and Editing: all authors.

**Acknowledgements:** The authors would like to thank the study participants and staff of the Seattle Colon Cancer Family Registry and the Hormones and Colon Cancer study (CORE Studies). They would also like to thank Kimon Divaris for helpful guidance on selecting periodontitis-associated SNPs and genes.

## Data Availability

Data underlying this article are available through dbGAP through the Oncoarray Consortium—Lung Cancer Studies dbGaP Study Accession: phs001273.v3.p2 at https://www.ncbi.nlm.nih.gov/projects/gap/cgi-bin/analysis.cgi?study_id=phs001273.v3.p2&phv=282571&phd=7215&pha=4930&pht=6171&phvf=&phdf=&phaf=&phtf=&dssp=1&consent=&temp=1; https://www.ncbi.nlm.nih.gov/projects/gap/cgi-bin/study.cgi?study_id=phs000206.v3.p2; https://www.ncbi.nlm.nih.gov/projects/gap/cgi-bin/study.cgi?study_id=phs000648.v1.p1; https://www.ncbi.nlm.nih.gov/projects/gap/cgi-bin/study.cgi?study_id=phs001078.v1.p1; and https://www.ncbi.nlm.nih.gov/projects/gap/cgi-bin/study.cgi?study_id=phs001499.v1.p1. Data unavailable in dbGaP can be requested from the respective consortia.

## Supplementary Material

pkab037_Supplementary_Data
