# Supplementary material for: Two-Sample Mendelian Randomization Analysis of Associations Between Periodontal Disease and Risk of Cancer
Source: JNCI Cancer Spectr. 2021 Apr 19;5(3):pkab037. doi: 10.1093/jncics/pkab037 (PMC8242136; doi:10.1093/jncics/pkab037)
Supplement: pkab037_Supplementary_Data [file pkab037_supplementary_data.pdf]

SUPPLEMENTARY MATERIAL

Supplementary Methods

Definition of periodontitis

Aggressive periodontitis was defined by percentage bone loss affecting multiple teeth in adults <36 years of age as determined by full-mouth dental radiographs.[1–4] Chronic and severe periodontitis were defined by measures such as age- and sex-specific groups of attachment loss ≥ 4 mm;[1,3] self-reported gum surgery;[5] or probing depth, clinical attachment level, plaque index, gingival index, and bleeding for multiple teeth.[5–7]

Primary genetic instrument for periodontal disease

We considered the evidence very strong for an association with periodontal disease if the association met the genome-wide significance threshold of $p < 5 \times 10^{-8}$ in a pooled analysis of multiple cohorts. Six SNPs (rs729876, rs1537415, rs2738058, rs2978951, rs4284742, and rs16870060) met this definition.[1–3] Four of these SNPs (rs729876, rs2738058, rs4284742, and rs16870060) were used as the instrumental variables for periodontitis in a previous MR study on hypertension; the authors did not include rs1537415 because it is palindromic and did not have a proxy SNP in Caucasians.[8] We included the palindromic rs1537415 in our primary analysis because the minor allele frequency was 0.42 and we considered this sufficiently different from 0.50; however, we also conducted sensitivity analyses excluding this allele. Since rs2738058 and rs2978951 were in linkage disequilibrium ($r^2 = 0.27$; $p < 0.001$ for European populations),[9] and since rs2738058 had a larger estimated effect estimate and smaller p-value (odds ratio = 1.28 versus 1.25; $p = 6.78 \times 10^{-10}$ versus 2.06×10^{-8}, respectively), we excluded rs2978951.[3] Similarly, we included rs12461706 instead of rs4284742 because the two SNPs were in linkage disequilibrium ($r^2 = 0.19$; $p < 0.001$ for European populations),[9] one study observed that rs12461706 was significantly associated with periodontitis and loose teeth ($p = 3.9 \times 10^{-9}$ in a combined analysis; consistent effect estimates observed in two study samples),[5] the p-values reported in

PhenoScanner for associations with dental health traits were smaller for rs12461706 than for rs4284742,[10] and substituting rs12461706 substantially increased the I^2_{GX} value (0.62 versus 0.93). In addition to the five SNPs with very strong evidence for an association, we identified three SNPs (rs2521634, rs3826782, and rs7762544) with strong evidence for an association. These SNPs were positively associated with chronic periodontitis in one cohort ($p < 5 \times 10^{-6}$), nominally positively associated ($p < 0.05$) with severe chronic periodontitis in an independent replication cohort, and positively associated ($p < 5 \times 10^{-6}$) with chronic periodontitis in a meta-analysis of over 5000 European American individuals in these cohorts.[6]

Secondary genetic instrument for periodontal disease

In addition to the eight SNPs included in the primary instrument, we identified seven SNPs with moderate evidence for an association with periodontitis. We considered the evidence moderate if the associations were significant at a threshold of $p < 5 \times 10^{-6}$ in a pooled analysis of multiple cohorts but not associated with periodontitis in any single cohort with a threshold of $p < 5 \times 10^{-6}$. Three of the SNPs that met this criteria (rs1122900, rs2070901, and rs4970469) were associated with aggressive periodontitis ($p < 5 \times 10^{-6}$) in the combined results from a discovery meta-analysis and replication cohort.[3] The other four SNPs (rs11084095, rs2064712, rs9982623, and rs9984417) were associated with aggressive and chronic periodontitis ($p < 5 \times 10^{-6}$) in pooled cohorts.[1] One of these SNPs (rs11084095) was in linkage disequilibrium with a SNP we identified as having very strong evidence for an association (rs12461706) so we excluded rs11084095 ($r^2 = 0.99$; $p < 0.001$ for European populations).[9]

Quality assurance measures for the SNPs included in the genetic instruments

We assessed the potential for pleiotropy, issues of SNPs being in mutual linkage disequilibrium, whether the GWAS accounted for population stratification, and issues of harmonization. Of the 14 SNPs, only rs2070901, rs2738058, and rs12461706 were associated ($p < 5 \times 10^{-8}$) with any diseases or traits other than periodontitis or other oral health measures.[11] Notably, the associated traits were primarily

related to inflammatory processes (e.g., white blood cell differential) that may be part of the putative causal pathways between periodontal disease and cancer risk (thus introducing vertical pleiotropy and potentially strengthening the genetic instrument). None of the included SNPs was in linkage disequilibrium with any other included SNP.[9] Similarly, population stratification seems unlikely to be a major confounder of the GWAS results; the participants included in the analyses for all of the SNPs are primarily from populations of European descent[1–3,6] and most of the studies explicitly considered population stratification.[3,5,6] All genetic variants were positively associated with periodontitis. We verified that the genetic variant was the same in each dataset for palindromic alleles (rs1537415, rs9984417, and rs12461706), and we conducted sensitivity analyses removing each of these SNPs (one at a time) to ensure that the effect estimates were not driven by issues related to harmonization.

Summary-level data for lung, colorectal, and pancreatic cancer

For GECCO, genotyping was performed with one of the Illumina 1536 GoldenGate assay (Illumina, Inc, San Diego, CA), the Affymetrix GeneChip Human Mapping 100K and 500K Array Set (Affymetrix, Inc, Santa Clara, CA), or a 10K nonsynonymous SNP chip.[12] SNPs were excluded from genotyping if the call rate was <98%, there was a low minor allele frequency (<1%), or there was a lack of Hardy-Weinberg equilibrium in controls ($p < 10^{-4}$). Imputed data were included for autosomal SNPs with minor allele frequency ≥1% and high imputation accuracy ($R^2 > 0.3$). The reference population included Utah residents of Northern and Western European ancestry from HapMap II.[13] The SNP-colorectal cancer estimates were adjusted for age, sex, and genetic principal components. For ILCCO, quality control procedures for genotyping with the OncoArray included steps to account for duplicates, related individuals, Hardy-Weinberg equilibrium, and call rates.[14] Imputation was conducted with the 1000 Genomes Project Phase 3 as a reference.[15] The SNP-lung cancer estimates were also adjusted for age, sex, and genetic principal components.[16] For PanScan I, II, and III, genotyping was performed at the National Cancer Institute Cancer Genomics Research Laboratory with Illumina HumanHap series arrays

(Illumina HumanHap550 Infinium II, Human 610-Quad) for PanScan I and II, respectively, and the Illumina Omni series arrays (OmniExpress, Omni1M, Omni2.5 and Omni5M) for PanScan III. Quality control procedures accounted for duplicates, related individuals, population admixture, Hardy-Weinberg equilibrium, and call rates. PanC4 was genotyped at the Center for Inherited Disease Research.[17] Genotyping was performed with the Illumina HumanOmniExpressExome-8v1 array.[18] Imputation for both PanScan and PanC4 was conducted using the 1000 Genomes Phase 3, Release 1 reference data set and IMPUTE2.[19–22] Imputation analyses were adjusted for age in decade, sex, and top eigenvectors (five for PanScan; nine for PanC4) from principal components analysis to control for ancestry. PanScan analyses were also adjusted for study and geographic region of the parent studies.

Explanation of the MR approaches

We used four MR approaches to assess the association of genetic predisposition index for having chronic or aggressive periodontal disease on colorectal, lung, and pancreatic cancer risk. Our primary method was the inverse-variance weighted (IVW) method, so named because it weights the SNPs by the inverse of the variance.[23] The IVW method is commonly used because it has greater power to detect associations than many other MR methods and because the results are equivalent to the two-stage least squares method that would be obtained using individual-level data. Since the IVW method assumes that each genetic variant is a valid instrumental variable and it forces a zero intercept in the regression slope, the IVW method assumes either no pleiotropy or balanced directional pleiotropy and that any balanced pleiotropic effects of a genetic variant are independent of the association with the exposure.[24–26]

Our three alternative MR methods were MR-PRESSO, simple median, and weighted median. MR-PRESSO identifies and excludes outlier SNPs using the residual sum of squares heterogeneity measure.[27] It evaluates associations using a three stage process based on the residual sum of squares using a leave-one-SNP-out approach.[28] Although this method is efficient if each SNP is a valid instrument

and it can help handle directional pleiotropy, it is prone to false positives if multiple SNPs are invalid.[24] The other two MR methods we considered (simple median and weighted median) assume that the majority of SNPs are valid.[24,26,29] The weighted median approach uses weights based on the ratio of the SNP-outcome to SNP-exposure[30] whereas the simple median approach uses the median ratio estimate for all of the SNPs.[31] We did not use MR-Egger (a common MR method that examines directional pleiotropy) because the consistency of MR-Egger effect estimates increases as the number of genetic variants increases.[32] Specifically, the MR-Egger regression models would not have sufficient power to detect true associations (with 15 SNPs, MR-Egger has power = 0.25 versus the IVW method that has power = 0.78).[32]

A note about the interpretation of MR results

Given that MR analyses inherently account for long-term influences of genetic variants, associations are more accurately interpreted as lifetime exposure to a predisposition of periodontal disease – rather than associations with periodontal disease itself. We note that the nuance in interpretation here is important, but perhaps less critical than in a MR study for time-varying exposures such as smoking behavior (where cumulative exposure is not well approximated by most measures of exposure). A second consideration for MR analyses is whether there are factors that affect survival of individuals with (or without) a genetic predisposition to the exposure (throughout life, until the time of recruitment) and also affect the likelihood of the outcome. In our analysis, we expect that any such survivor bias would be minimal and would bias the effect estimates towards the null.

References

1. Munz M, Richter GM, Loos BG, et al. Meta-analysis of genome-wide association studies of aggressive and chronic periodontitis identifies two novel risk loci. *Eur J Hum Genet*. 2019;27(1):102-113. doi:10.1038/s41431-018-0265-5

2. Schaefer AS, Richter GM, Nothnagel M, et al. A genome-wide association study identifies GLT6D1 as a susceptibility locus for periodontitis. *Hum Mol Genet*. 2010;19(3):553-562. doi:10.1093/hmg/ddp508

3. Munz M, Willenborg C, Richter GM, et al. A genome-wide association study identifies nucleotide variants at SIGLEC5 and DEFA1A3 as risk loci for periodontitis. *Hum Mol Genet*. 2017;26(13):2577-2588. doi:10.1093/hmg/ddx151

4. Schaefer AS, Richter GM, Groessner-Schreiber B, et al. Identification of a Shared Genetic Susceptibility Locus for Coronary Heart Disease and Periodontitis. *PLOS Genetics*. 2009;5(2):e1000378. doi:10.1371/journal.pgen.1000378

5. Shungin D, Haworth S, Divaris K, et al. Genome-wide analysis of dental caries and periodontitis combining clinical and self-reported data. *Nat Commun*. 2019;10(1):2773. doi:10.1038/s41467-019-10630-1

6. Divaris K, Monda KL, North KE, et al. Exploring the genetic basis of chronic periodontitis: a genome-wide association study. *Hum Mol Genet*. 2013;22(11):2312-2324. doi:10.1093/hmg/ddt065

7. Page RC, Eke PI. Case Definitions for Use in Population-Based Surveillance of Periodontitis. *Journal of Periodontology*. 2007;78(7S):1387-1399. doi:10.1902/jop.2007.060264

8. Czesnikiewicz-Guzik M, Osmenda G, Siedlinski M, et al. Causal association between periodontitis and hypertension: evidence from Mendelian randomization and a randomized controlled trial of non-surgical periodontal therapy. *Eur Heart J*. 2019;40(42):3459-3470. doi:10.1093/eurheartj/ehz646

9. National Cancer Institute. LDpair Tool | An Interactive Web Tool for Exploring Linkage Disequilibrium in Population Groups. Accessed August 1, 2020. https://ldlink.nci.nih.gov/?tab=ldpair

10. University of Cambridge. PhenoScanner v2: rs12461706. Accessed August 6, 2020. http://www.phenoscanner.medschl.cam.ac.uk/?query=rs12461706&catalogue=GWAS&p=1e-5&proxies=None&r2=0.8&build=37

11. University of Cambridge. PhenoScanner v2. Accessed August 1, 2020. http://www.phenoscanner.medschl.cam.ac.uk/

12. Peters U, Jiao S, Schumacher FR, et al. Identification of Genetic Susceptibility Loci for Colorectal Tumors in a Genome-Wide Meta-analysis. *Gastroenterology*. 2013;144(4):799-807.e24. doi:10.1053/j.gastro.2012.12.020

13. International HapMap Consortium. A second generation human haplotype map of over 3.1 million SNPs. *Nature*. 2007;449(7164):851-861. doi:10.1038/nature06258

14. Amos CI, Dennis J, Wang Z, et al. The OncoArray Consortium: a Network for Understanding the Genetic Architecture of Common Cancers. *Cancer Epidemiol Biomarkers Prev*. 2017;26(1):126-135. doi:10.1158/1055-9965.EPI-16-0106

15. International Genome Sample Resource. Data portal. Accessed August 1, 2020. https://www.internationalgenome.org/data/

16. McKay JD, Hung RJ, Han Y, et al. Large-scale association analysis identifies new lung cancer susceptibility loci and heterogeneity in genetic susceptibility across histological subtypes. *Nat Genet*. 2017;49(7):1126-1132. doi:10.1038/ng.3892

17. Wolpin BM, Rizzato C, Kraft P, et al. Genome-wide association study identifies multiple susceptibility loci for pancreatic cancer. *Nature Genetics*. 2014;46(9):994-1000. doi:10.1038/ng.3052

18. Klein AP, Wolpin BM, Risch HA, et al. Genome-wide meta-analysis identifies five new susceptibility loci for pancreatic cancer. *Nature Communications*. 2018;9(1):556. doi:10.1038/s41467-018-02942-5

19. Durbin RM, Altshuler D, Durbin RM, et al. A map of human genome variation from population-scale sequencing. *Nature*. 2010;467(7319):1061-1073. doi:10.1038/nature09534

20. IMPUTE2. Accessed September 8, 2020. http://mathgen.stats.ox.ac.uk/impute/impute_v2.html

21. Marchini J, Howie B, Myers S, McVean G, Donnelly P. A new multipoint method for genome-wide association studies by imputation of genotypes. *Nature Genetics*. 2007;39(7):906-913. doi:10.1038/ng2088

22. Wang Z, Jacobs KB, Yeager M, et al. Improved Imputation of Common and Uncommon Single Nucleotide Polymorphisms (SNPs) with a New Reference Set. *Nat Genet*. 2011;44(1):6-7. doi:10.1038/ng.1044

23. Burgess S, Butterworth A, Thompson SG. Mendelian Randomization Analysis With Multiple Genetic Variants Using Summarized Data. *Genetic Epidemiology*. 2013;37(7):658-665. doi:https://doi.org/10.1002/gepi.21758

24. Slob EAW, Burgess S. A comparison of robust Mendelian randomization methods using summary data. *Genetic Epidemiology*. 2020;44(4):313-329. doi:10.1002/gepi.22295

25. Hwang L-D, Lawlor DA, Freathy RM, Evans DM, Warrington NM. Using a two-sample Mendelian randomization design to investigate a possible causal effect of maternal lipid concentrations on offspring birth weight. *Int J Epidemiol*. 2019;48(5):1457-1467. doi:10.1093/ije/dyz160

26. Bowden J, Davey Smith G, Haycock PC, Burgess S. Consistent Estimation in Mendelian Randomization with Some Invalid Instruments Using a Weighted Median Estimator. *Genet Epidemiol*. 2016;40(4):304-314. doi:10.1002/gepi.21965

27. Verbanck M, Chen C-Y, Neale B, Do R. Detection of widespread horizontal pleiotropy in causal relationships inferred from Mendelian randomization between complex traits and diseases. *Nat Genet*. 2018;50(5):693-698. doi:10.1038/s41588-018-0099-7

28. Ong J-S, MacGregor S. Implementing MR-PRESSO and GCTA-GSMR for pleiotropy assessment in Mendelian randomization studies from a practitioner's perspective. *Genetic Epidemiology*. 2019;43(6):609-616. doi:https://doi.org/10.1002/gepi.22207

29. Hartwig FP, Davey Smith G, Bowden J. Robust inference in summary data Mendelian randomization via the zero modal pleiotropy assumption. *Int J Epidemiol*. 2017;46(6):1985-1998. doi:10.1093/ije/dyx102

30. Hwang L-D, Lawlor DA, Freathy RM, Evans DM, Warrington NM. Using a two-sample Mendelian randomization design to investigate a possible causal effect of maternal lipid concentrations on offspring birth weight. *International Journal of Epidemiology*. 2019;48(5):1457-1467. doi:10.1093/ije/dyz160

31. Bowden J, Davey Smith G, Haycock PC, Burgess S. Consistent Estimation in Mendelian Randomization with Some Invalid Instruments Using a Weighted Median Estimator. *Genet Epidemiol*. 2016;40(4):304-314. doi:10.1002/gepi.21965

32. Bowden J, Davey Smith G, Burgess S. Mendelian randomization with invalid instruments: effect estimation and bias detection through Egger regression. *Int J Epidemiol*. 2015;44(2):512-525. doi:10.1093/ije/dyv080

Supplementary Table 1. Single nucleotide polymorphisms (SNPs) used as instrumental variables based on their association with periodontal disease

Evidence	rsID	Chromosome	Nearest gene (or pseudogene)	Effect allele	Non-effect allele	OR (95% CI)	Effect allele frequency (periodontal disease)	Effect allele frequency (colorectal cancer, lung cancer, and pancreatic cancer)	Periodontal disease definition[5] and reference number
Very strong[1]	rs729876	16	*SHISA9*	T	C	1.24 (1.15-1.34)	0.79-0.84	0.80-0.81	Aggressive or chronic (1)
	rs1537415	9	*GLT6D1*	G	C	1.59 (1.36-1.86)	0.50-0.62	0.59-0.60	Aggressive (2)
	rs2738058	8	*DEFA1A3*	T	C	1.28 (1.18-1.38)	0.43	0.42-0.44	Aggressive or chronic (3)
	rs12461706	19	*SIGLEC5*	T	A	1.05 (1.03, 1.07)[4]	0.40	0.39-0.41	Chronic (5)
	rs16870060	8	*MTND1P5*	G	T	1.36 (1.23-1.51)	0.89-0.94	0.91	Aggressive or chronic (1)
Strong[2]	rs2521634	7	*NPY*	A	G	1.49 (1.28-1.73)	0.20-0.26	0.24	Chronic (6)
	rs3826782	19	*EMR1*	A	G	2.01 (1.52-2.65)	0.04-0.05	0.09-0.11	Chronic (6)
	rs7762544	6	*NCR2*	G	A	1.40 (1.24-1.59)	0.16-0.21	0.19-0.20	Chronic (6)
Moderate[3]	rs1122900	5	*CTD-2353F22.1*	A	C	1.27 (1.16-1.40)	0.40	0.42	Aggressive or chronic (3)
	rs2064712	6	*AL109933.3-AL391361.2*	A	G	1.24 (1.14-1.35)	0.09-0.19	0.11-0.16	Aggressive or chronic (1)
	rs2070901	1	*FCER1G*	T	G	1.29 (1.16-1.44)	0.24	0.26-0.27	Aggressive or chronic (3)
	rs4970469	1	*OSTCP2*	G	A	1.52 (1.29-1.81)	0.90	0.89-0.90	Aggressive or chronic (3)
	rs9982623	21	*MCM3AP*	C	T	1.24 (1.14-1.35)	0.86-0.89	0.86-0.88	Aggressive or chronic (1)
	rs9984417	21	*MAPK6PS2-AP000959.2*	T	A	1.15 (1.09-1.23)	0.61-0.68	0.60-0.61	Aggressive or chronic (1)

[1]Very strong evidence: the association with periodontitis met the genome-wide significance threshold of $p < 5 \times 10^{-8}$ in a pooled analysis of multiple cohorts (for each level of evidence, focusing on populations of European descent to match the population demographics of our outcome data)

[2]Strong evidence: associated with periodontitis in at least one cohort with a threshold of $p < 5 \times 10^{-6}$, same direction of association in an independent cohort with a threshold of $p < 0.05$, same direction of association in a meta-analysis of these cohorts with a threshold of $p < 5 \times 10^{-6}$

[3]Moderate evidence: associated with periodontitis with a threshold of $p < 5 \times 10^{-6}$ in a pooled analysis of multiple cohorts but not associated with periodontitis in any single cohort with a threshold of $p < 5 \times 10^{-6}$

[4]The 95% confidence interval for this SNP was calculated using the p-value and sample size reported in the source article.

[5]Aggressive periodontitis was defined by percentage bone loss affecting multiple teeth in adults <36 years of age as determined by full-mouth dental radiographs.[1–4] Chronic and severe periodontitis were defined by measures such as age- and sex-specific groups of attachment loss ≥ 4 mm;[1,3] self-reported gum surgery;[5] or probing depth, clinical attachment level, plaque index, gingival index, and bleeding for multiple teeth.[5–7]

Supplementary Table 2. Effect estimates for the association between genetic predisposition to having chronic or aggressive periodontitis and the risk of colorectal, lung, and pancreatic cancer by MR approach and genetic instrument sensitivity analysis.

**Outcome**	**Instrument[1]**	**IVW[2] β (p-value)**	**MR-PRESSO β (p-value)**	**Simple median β (p-value)**	**Weighted median β (p-value)**
Colorectal (N_{cases}= 58131, $N_{controls}$ = 67347)	Primary	0.025 (0.026)	0.025 (0.010)	0.025 (0.115)	0.027 (0.063)
	Primary excluding rs1537415	0.024 (0.077)	0.024 (0.041)	0.023 (0.246)	0.027 (0.114)
	Primary excluding rs3826782	0.031 (0.012)	0.031 (0.002)	0.027 (0.137)	0.030 (0.052)
	Primary excluding rs12461706	0.025 (0.025)	0.025 (0.015)	0.027 (0.090)	0.027 (0.063)
	Primary excluding rs1537415 and rs12461706	0.024 (0.074)	0.024 (0.058)	0.029 (0.094)	0.027 (0.105)
	Secondary	0.006 (0.699)	0.016 (0.106)	0.002 (0.875)	0.025 (0.054)
	Secondary excluding rs9984417	0.006 (0.692)	0.017 (0.111)	0.007 (0.682)	0.025 (0.057)
Lung (N_{cases} = 18082, $N_{controls}$ = 13780)	Primary	0.004 (0.832)	0.004 (0.762)	-0.020 (0.477)	0.019 (0.446)
	Primary excluding rs1537415	-0.003 (0.878)	-0.003 (0.829)	-0.035 (0.320)	0.001 (0.966)
	Primary excluding rs3826782	-0.001 (0.950)	-0.001 (0.932)	-0.035 (0.330)	0.003 (0.905)
	Primary excluding rs12461706	0.005 (0.809)	0.005 (0.742)	-0.006 (0.833)	0.019 (0.440)
	Primary excluding rs1537415 and rs12461706	-0.003 (0.904)	-0.003 (0.873)	-0.020 (0.505)	0.001 (0.961)
	Secondary	-0.006 (0.745)	-0.006 (0.750)	-0.011 (0.655)	0.017 (0.449)
	Secondary excluding rs9984417	-0.007 (0.704)	-0.007 (0.711)	-0.017 (0.532)	0.015 (0.496)
Pancreatic (PanScan) (N_{cases} = 5090, $N_{controls}$ = 8733)	Primary	0.031 (0.372)	0.031 (0.189)	0.031 (0.537)	0.026 (0.550)
	Primary excluding rs1537415	0.032 (0.428)	0.032 (0.276)	0.035 (0.583)	0.031 (0.561)
	Primary excluding rs3826782	0.037 (0.344)	0.037 (0.195)	0.035 (0.574)	0.033 (0.516)
	Primary excluding rs12461706	0.031 (0.364)	0.031 (0.215)	0.035 (0.471)	0.026 (0.545)
	Primary excluding rs1537415 and rs12461706	0.033 (0.418)	0.033 (0.309)	0.044 (0.416)	0.031 (0.550)
	Secondary	0.033 (0.262)	0.033 (0.170)	0.044 (0.312)	0.026 (0.513)
	Secondary excluding rs9984417	0.033 (0.262)	0.033 (0.170)	0.044 (0.312)	0.026 (0.513)
Pancreatic (PanC4) (N_{cases} = 4164, $N_{controls}$ = 3792)	Primary	0.000 (0.994)	0.000 (0.992)	-0.020 (0.738)	-0.030 (0.548)
	Primary excluding rs1537415	0.020 (0.665)	0.020 (0.568)	0.008 (0.913)	0.012 (0.843)
	Primary excluding rs3826782	0.017 (0.708)	0.017 (0.620)	0.008 (0.912)	0.002 (0.970)
	Primary excluding rs12461706	0.000 (0.991)	0.000 (0.989)	0.008 (0.893)	-0.029 (0.550)
	Primary excluding rs1537415 and rs12461706	0.021 (0.648)	0.021 (0.577)	0.031 (0.613)	0.012 (0.834)
	Secondary	0.006 (0.855)	0.006 (0.785)	0.030 (0.551)	-0.042 (0.353)

	Secondary excluding rs9984417	0.006 (0.855)	0.006 (0.785)	0.030 (0.551)	-0.042 (0.353)

[1]The primary analysis included eight SNPs (rs729876, rs1537415, rs2738058, rs12461706, rs16870060, rs2521634, rs3826782, and rs7762544). The secondary analysis included six additional SNPs (rs1122900, rs2064712, rs2070901, rs4970469, rs9982623, and rs9984417).

[2]The primary Mendelian randomization methods was inverse-variance weighted (IVW) MR. We used MR-PRESSO, simple median, and weighted median as secondary analyses. Betas indicate the effect estimate for the association between a one-unit increase in genetic predisposition to having chronic or aggressive periodontitis and the natural log risk for each outcome.

Supplementary Table 3. Effect estimates for the association between genetic predisposition to having chronic or aggressive periodontitis and the risk of colorectal cancer by histologic type, sex, genetic instrument, and MR approach.

Outcome	N_{cases}/ $N_{controls}$	Instrument[1]	IVW[2] β (p-value)	MR-PRESSO β (p-value)	Simple median β (p-value)	Weighted median β (p-value)
All colorectal cancer	58131/ 67347	Primary	0.025 (0.026)	0.025 (0.010)	0.025 (0.115)	0.027 (0.063)
		Secondary	0.006 (0.699)	0.016 (0.106)	0.002 (0.875)	0.025 (0.054)
Colon cancer	31083/ 67347	Primary	0.031 (0.021)	0.031 (0.000)	0.030 (0.101)	0.030 (0.075)
		Secondary	0.010 (0.469)	0.010 (0.481)	0.017 (0.336)	0.027 (0.070)
Rectal cancer	15775/ 67347	Primary	0.002 (0.933)	0.002 (0.936)	-0.015 (0.550)	0.011 (0.644)
		Secondary	-0.014 (0.472)	-0.014 (0.485)	-0.043 (0.093)	0.004 (0.866)
Distal cancer	15306/ 67347	Primary	0.023 (0.190)	0.023 (0.026)	0.013 (0.593)	0.027 (0.215)
		Secondary	-0.001 (0.972)	-0.001 (0.973)	-0.009 (0.707)	0.014 (0.471)
Proximal cancer	13857/ 67347	Primary	0.043 (0.013)	0.043 (0.002)	0.027 (0.279)	0.036 (0.100)
		Secondary	0.020 (0.212)	0.020 (0.234)	0.020 (0.360)	0.029 (0.139)
Colorectal cancer in females	26843/ 32820	Primary	0.033 (0.039)	0.033 (0.065)	0.052 (0.033)	0.045 (0.037)
		Secondary	0.016 (0.333)	0.016 (0.351)	0.012 (0.574)	0.031 (0.104)
Colorectal cancer in males	31288/ 34527	Primary	0.021 (0.174)	0.021 (0.010)	0.021 (0.320)	0.021 (0.266)
		Secondary	-0.002 (0.923)	-0.002 (0.924)	0.007 (0.731)	0.016 (0.384)

[1]The primary analysis included eight SNPs (rs729876, rs1537415, rs2738058, rs12461706, rs16870060, rs2521634, rs3826782, and rs7762544). The secondary analysis included six additional SNPs (rs1122900, rs2064712, rs2070901, rs4970469, rs9982623, and rs9984417).

[2]The primary Mendelian randomization methods was inverse-variance weighted (IVW) MR. We used MR-PRESSO, simple median, and weighted median as secondary analyses. Betas indicate the effect estimate for the association between a one-unit increase in genetic predisposition to having chronic or aggressive periodontitis and the natural log risk for each outcome.

Supplementary Table 4. Effect estimates for the association between genetic predisposition to having chronic or aggressive periodontitis and the risk of lung cancer by cancer location, smoker status, genetic instrument, and MR approach.

Outcome	N_{cases}/ $N_{controls}$	Instrument[1]	IVW[2] β (p-value)	MR-PRESSO β (p-value)	Simple median β (p-value)	Weighted median β (p-value)
Adenocarcinoma	6730/ 13780	Primary	0.025 (0.313)	0.025 (0.248)	0.035 (0.320)	0.036 (0.266)
		Secondary	0.012 (0.708)	0.031 (0.222)	0.040 (0.201)	0.038 (0.196)
Squamous cell	4429/ 13780	Primary	-0.010 (0.740)	-0.010 (0.706)	-0.028 (0.498)	0.011 (0.769)
		Secondary	-0.019 (0.446)	-0.019 (0.449)	-0.028 (0.454)	0.008 (0.807)
Small cell	1853/ 13780	Primary	-0.035 (0.400)	-0.035 (0.205)	-0.033 (0.602)	-0.033 (0.545)
		Secondary	-0.019 (0.589)	-0.019 (0.416)	-0.032 (0.549)	-0.034 (0.486)
Smokers	15984/ 9084	Primary	0.002 (0.927)	0.002 (0.924)	0.011 (0.758)	0.020 (0.506)
		Secondary	0.001 (0.972)	0.001 (0.970)	0.011 (0.715)	0.018 (0.519)
Non-smokers	1800/ 4415	Primary	-0.021 (0.652)	-0.021 (0.615)	-0.051 (0.511)	0.010 (0.885)
		Secondary	-0.042 (0.394)	-0.042 (0.409)	-0.022 (0.746)	0.006 (0.915)
Smokers with adenocarcinoma	5639/ 9084	Primary	0.029 (0.317)	0.029 (0.350)	0.034 (0.418)	0.042 (0.274)
		Secondary	0.018 (0.513)	0.018 (0.525)	0.034 (0.356)	0.041 (0.239)
Non-smokers with adenocarcinoma	975/ 4415	Primary	0.012 (0.839)	0.012 (0.829)	0.054 (0.582)	0.072 (0.387)
		Secondary	0.008 (0.916)	0.053 (0.422)	0.114 (0.182)	0.103 (0.193)
Smokers with squamous cell	4209/ 9084	Primary	-0.019 (0.542)	-0.019 (0.554)	-0.084 (0.092)	0.003 (0.938)
		Secondary	-0.012 (0.679)	-0.012 (0.686)	-0.060 (0.146)	0.002 (0.952)
Non-smokers with squamous cell	158/ 4415	Primary	-0.040 (0.773)	-0.040 (0.703)	0.058 (0.777)	0.034 (0.849)
		Secondary	-0.217 (0.070)	-0.217 (0.069)	-0.342 (0.091)	-0.256 (0.130)
Smokers with small cell	1761/ 9084	Primary	-0.023 (0.593)	-0.023 (0.547)	0.010 (0.884)	-0.003 (0.954)
		Secondary	0.001 (0.974)	0.001 (0.967)	0.027 (0.649)	-0.006 (0.910)
		Primary	-0.383 (0.059)	-0.383 (0.052)	-0.329 (0.243)	-0.356 (0.170)

Non-smokers with small cell	64/ 4415	Secondary	-0.412 (0.021)	-0.412 (0.017)	-0.352 (0.163)	-0.361 (0.131)

[1]The primary analysis included eight SNPs (rs729876, rs1537415, rs2738058, rs12461706, rs16870060, rs2521634, rs3826782, and rs7762544). The secondary analysis included six additional SNPs (rs1122900, rs2064712, rs2070901, rs4970469, rs9982623, and rs9984417).

[2]The primary Mendelian randomization methods was inverse-variance weighted (IVW) MR. We used MR-PRESSO, simple median, and weighted median as secondary analyses. Betas indicate the effect estimate for the association between a one-unit increase in genetic predisposition to having chronic or aggressive periodontitis and the natural log risk for each outcome.

Supplementary Table 5. Effect estimates for the association between genetic predisposition to having chronic or aggressive periodontitis and the risk of pancreatic cancer in the PanScan meta-analysis by sex, smoker status, study design, genetic instrument, and Mendelian randomization approach.

Study design	Sub-set	N_{cases} / $N_{controls}$	Instrument[1]	IVW[2] β (p-value)	MR-PRESSO[2] β (p-value)	Simple median β (p-value)	Weighted median β (p-value)
All	All	5090/8733	primary	0.031 (0.372)	0.031 (0.189)	0.031 (0.537)	0.026 (0.550)
			secondary	0.033 (0.262)	0.033 (0.170)	0.044 (0.312)	0.026 (0.513)
	Female	2475/3048	primary	0.020 (0.691)	0.020 (0.703)	-0.018 (0.803)	0.017 (0.787)
			secondary	0.007 (0.877)	0.007 (0.856)	-0.036 (0.567)	0.015 (0.794)
	Male	2615/5685	primary	0.045 (0.347)	0.045 (0.305)	0.071 (0.318)	0.011 (0.858)
			secondary	0.067 (0.107)	0.067 (0.112)	0.115 (0.077)	0.014 (0.804)
	Current smoker	874/1286	primary	0.069 (0.437)	0.069 (0.269)	0.046 (0.720)	0.031 (0.784)
			secondary	0.034 (0.664)	0.034 (0.581)	0.040 (0.725)	0.030 (0.771)
	Former smoker	1871/3676	primary	0.053 (0.451)	0.053 (0.476)	0.033 (0.703)	0.064 (0.399)
			secondary	0.074 (0.129)	0.074 (0.149)	0.071 (0.322)	0.114 (0.086)
	Never smoker	1735/3388	primary	-0.023 (0.692)	-0.023 (0.430)	0.009 (0.909)	-0.017 (0.819)
			secondary	-0.024 (0.631)	-0.024 (0.535)	0.009 (0.898)	-0.019 (0.779)
Cohort	All	1367/5120	primary	0.108 (0.117)	0.108 (0.052)	0.078 (0.430)	0.100 (0.255)
			secondary	0.139 (0.020)	0.139 (0.012)	0.141 (0.114)	0.124 (0.121)
	Female	705/1431	primary	0.123 (0.248)	0.123 (0.286)	0.128 (0.387)	0.032 (0.805)
			secondary	0.156 (0.072)	0.156 (0.080)	0.164 (0.213)	0.038 (0.749)
	Male	662/3689	primary	0.078 (0.425)	0.078 (0.322)	0.063 (0.654)	0.159 (0.211)
			secondary	0.112 (0.187)	0.112 (0.103)	0.196 (0.116)	0.193 (0.095)
	Current smoker	367/850	primary	0.087 (0.523)	0.087 (0.468)	-0.097 (0.643)	0.037 (0.838)
			secondary	0.127 (0.286)	0.127 (0.210)	0.021 (0.907)	0.063 (0.698)
	Former smoker	485/2332	primary	0.128 (0.322)	0.128 (0.355)	0.057 (0.755)	0.067 (0.670)
			secondary	0.173 (0.106)	0.173 (0.130)	0.037 (0.814)	0.079 (0.574)
	Never smoker	461/1930	primary	0.036 (0.762)	0.036 (0.768)	0.109 (0.536)	-0.022 (0.891)
			secondary	0.075 (0.464)	0.075 (0.471)	0.130 (0.421)	-0.021 (0.887)
Case-control	All	2537/2371	primary	0.039 (0.480)	0.039 (0.263)	0.078 (0.333)	0.055 (0.437)
			secondary	0.006 (0.900)	0.006 (0.870)	0.076 (0.282)	0.051 (0.418)
	Female	1148/956	primary	0.034 (0.679)	0.034 (0.671)	-0.014 (0.915)	0.047 (0.674)
			secondary	-0.039 (0.582)	-0.039 (0.590)	-0.089 (0.428)	-0.056 (0.577)
	Male	1389/1415	primary	0.042 (0.567)	0.042 (0.329)	0.041 (0.689)	0.037 (0.691)

			secondary	0.047 (0.459)	0.047 (0.412)	0.041 (0.653)	0.035 (0.682)
	Current smoker	318/262	primary	0.125 (0.486)	0.125 (0.477)	0.244 (0.400)	0.192 (0.446)
			secondary	-0.005 (0.976)	-0.005 (0.976)	0.128 (0.609)	0.211 (0.350)
	Former smoker	1073/989	primary	0.065 (0.444)	0.065 (0.433)	0.060 (0.655)	0.136 (0.211)
			secondary	0.068 (0.347)	0.068 (0.223)	0.108 (0.311)	0.146 (0.127)
	Never smoker	903/1021	primary	-0.010 (0.906)	-0.010 (0.854)	-0.017 (0.897)	-0.037 (0.733)
			secondary	-0.061 (0.403)	-0.061 (0.278)	-0.077 (0.478)	-0.071 (0.473)

[1]The primary analysis included eight SNPs (rs729876, rs1537415, rs2738058, rs12461706, rs16870060, rs2521634, rs3826782, and rs7762544). The secondary analysis included six additional SNPs (rs1122900, rs2064712, rs2070901, rs4970469, rs9982623, and rs9984417).

[2]The primary Mendelian randomization methods was inverse-variance weighted (IVW) MR. We used MR-PRESSO, simple median, and weighted median as secondary analyses. Betas indicate the effect estimate for the association between a one-unit increase in genetic predisposition to having chronic or aggressive periodontitis and the natural log risk for each outcome.

Supplementary Table 6. Effect estimates for the association between genetic predisposition to having chronic or aggressive periodontitis and the risk of pancreatic cancer in PanC4 by sex, smoker status, genetic instrument, and Mendelian randomization approach.

**Outcome**	**N_{cases} / $N_{controls}$**	**Instrument[1]**	**IVW[2] β (p-value)**	**MR-PRESSO β (p-value)**	**Simple median β (p-value)**	**Weighted median β (p-value)**
Overall	4164/3792	Primary	0.000 (0.994)	0.000 (0.992)	-0.020 (0.738)	-0.030 (0.548)
		Secondary	0.006 (0.855)	0.006 (0.785)	0.030 (0.551)	-0.042 (0.353)
Male	2396/2106	Primary	-0.045 (0.432)	-0.045 (0.457)	-0.028 (0.747)	-0.053 (0.448)
		Secondary	-0.039 (0.388)	-0.039 (0.347)	-0.039 (0.574)	-0.056 (0.372)
Female	1768/1686	Primary	0.057 (0.338)	0.057 (0.305)	-0.022 (0.811)	0.087 (0.278)
		Secondary	0.063 (0.218)	0.063 (0.113)	0.047 (0.539)	0.118 (0.097)
Current smoker	643/438	Primary	0.030 (0.783)	0.030 (0.662)	-0.036 (0.814)	0.032 (0.811)
		Secondary	0.020 (0.831)	0.020 (0.793)	-0.036 (0.788)	-0.002 (0.985)
Former smoker	1415/1306	Primary	-0.093 (0.164)	-0.093 (0.075)	-0.099 (0.320)	-0.110 (0.208)
		Secondary	-0.031 (0.590)	-0.031 (0.556)	-0.021 (0.822)	-0.099 (0.223)
Never smoker	1579/1811	Primary	0.067 (0.310)	0.067 (0.343)	0.229 (0.046)	-0.008 (0.921)
		Secondary	0.058 (0.260)	0.058 (0.232)	0.057 (0.491)	0.016 (0.828)

[1]The primary analysis included eight SNPs (rs729876, rs1537415, rs2738058, rs12461706, rs16870060, rs2521634, rs3826782, and rs7762544). The secondary analysis included six additional SNPs (rs1122900, rs2064712, rs2070901, rs4970469, rs9982623, and rs9984417).

[2]The primary Mendelian randomization methods was inverse-variance weighted (IVW) MR. We used MR-PRESSO, simple median, and weighted median as secondary analyses. Betas indicate the effect estimate for the association between a one-unit increase in genetic predisposition to having chronic or aggressive periodontitis and the natural log risk for each outcome.

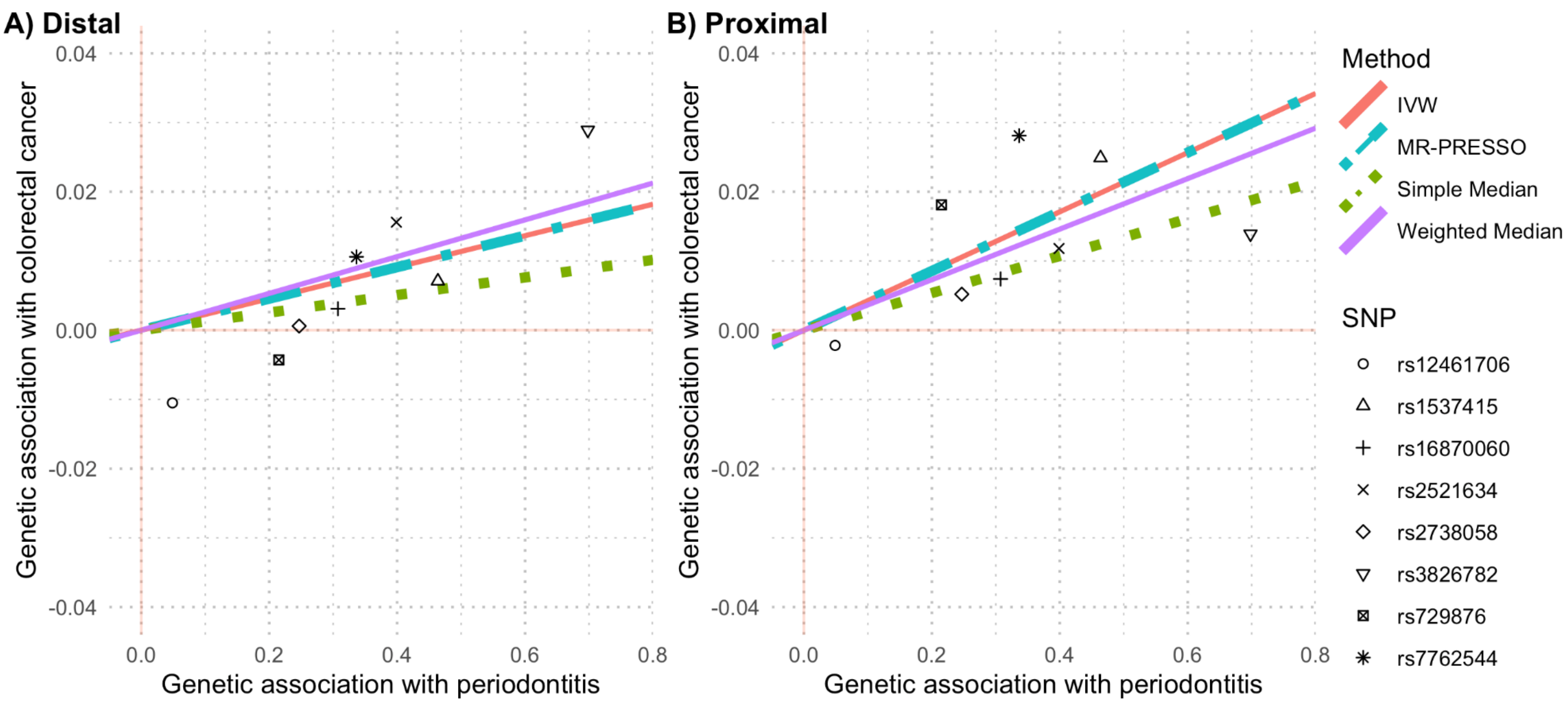

Supplementary Figure 1. Scatterplots comparing the strength of the SNP-exposure (periodontitis) and SNP-colorectal cancer associations. The lines indicate the estimated effect sizes by four Mendelian randomization methods (inverse-variance weighted (IVW), MR-PRESSO, simple median, and weighted median).

Funding and acknowledgments for the cancer consortia that provided genetic data for the analysis

Funding

The pancreatic cancer consortia were supported by the NIH grants R21 CA234436, U19CA203654, and K12HD092535.

Genetics and Epidemiology of Colorectal Cancer Consortium (GECCO): National Cancer Institute, National Institutes of Health, U.S. Department of Health and Human Services (U01 CA164930, U01 CA137088, R01 CA059045, R01201407, R01CA189532).Genotyping/Sequencing services were provided by the Center for Inherited Disease Research (CIDR). CIDR is fully funded through a federal contract from the National Institutes of Health to The Johns Hopkins University, contract number HHSN268201200008I. This research was funded in part through the NIH/NCI Cancer Center Support Grant P30 CA015704.

ASTERISK: a Hospital Clinical Research Program (PHRC-BRD09/C) from the University Hospital Center of Nantes (CHU de Nantes) and supported by the Regional Council of Pays de la Loire, the Groupement des Entreprises Françaises dans la Lutte contre le Cancer (GEFLUC), the Association Anne de Bretagne Génétique and the Ligue Régionale Contre le Cancer (LRCC).

The ATBC Study is supported by the Intramural Research Program of the U.S. National Cancer Institute, National Institutes of Health, and by U.S. Public Health Service contract HHSN261201500005C from the National Cancer Institute, Department of Health and Human Services.

CLUE funding was from the National Cancer Institute (U01 CA86308, Early Detection Research Network; P30 CA006973), National Institute on Aging (U01 AG18033), and the American Institute for Cancer Research. The content of this publication does not necessarily reflect the views or policies of the Department of Health and Human Services, nor does mention of trade names, commercial products, or organizations imply endorsement by the US government.COLO2&3: National Institutes of Health (R01 CA60987).

ColoCare: This work was supported by the National Institutes of Health (grant numbers R01 CA189184 (Li/Ulrich), U01 CA206110 (Ulrich/Li/Siegel/Figueireido/Colditz, 2P30CA015704- 40 (Gilliland), R01 CA207371 (Ulrich/Li)), the Matthias Lackas-Foundation, the German Consortium for Translational Cancer Research, and the EU TRANSCAN initiative.

The Colon Cancer Family Registry (CCFR, www.coloncfr.org) is supported in part by funding from the National Cancer Institute (NCI), National Institutes of Health (NIH) (award U01 CA167551). The CCFR Set-1 (Illumina 1M/1M-Duo) and Set-2 (Illumina Omni1-Quad) scans were supported by NIH awards U01 CA122839 and R01 CA143247 (to GC). The CCFR Set-3 (Affymetrix Axiom CORECT Set array) was supported by NIH award U19 CA148107 and R01 CA81488 (to SBG). The CCFR Set-4 (Illumina OncoArray 600K SNP array) was supported by NIH award U19 CA148107 (to SBG) and by the Center for Inherited Disease Research (CIDR), which is funded by the NIH to the Johns Hopkins University, contract number HHSN268201200008I. The content of this manuscript does not necessarily reflect the views or policies of the NCI, NIH or any of the collaborating centers in the Colon Cancer Family Registry (CCFR), nor does mention of trade names, commercial products, or organizations imply endorsement by the US Government, any cancer registry, or the CCFR.

COLON: The COLON study is sponsored by Wereld Kanker Onderzoek Fonds, including funds from grant 2014/1179 as part of the World Cancer Research Fund International Regular Grant Programme, by Alpe d'Huzes and the Dutch Cancer Society (UM 2012–5653, UW 2013-5927, UW2015-7946), and by TRANSCAN (JTC2012-MetaboCCC, JTC2013-FOCUS). The Nqplus study is sponsored by a ZonMW investment grant (98-10030); by PREVIEW, the project PREVention of diabetes through lifestyle intervention and population studies in Europe and around the World (PREVIEW) project which received funding from the European Union Seventh Framework Programme (FP7/2007–2013) under grant no. 312057; by funds from TI Food and Nutrition (cardiovascular health theme), a public–private partnership on precompetitive research in food and nutrition; and by FOODBALL, the Food Biomarker Alliance, a project from JPI Healthy Diet for a Healthy Life.

Colorectal Cancer Transdisciplinary (CORECT) Study: The CORECT Study was supported by the National Cancer Institute, National Institutes of Health (NCI/NIH), U.S. Department of Health and Human Services (grant numbers U19 CA148107, R01 CA81488, P30 CA014089, R01 CA197350,; P01 CA196569; R01 CA201407) and National Institutes of Environmental Health Sciences, National Institutes of Health (grant number T32 ES013678).

CORSA: "Österreichische Nationalbank Jubiläumsfondsprojekt" (12511) and Austrian Research Funding Agency (FFG) grant 829675.

CPS-II: The American Cancer Society funds the creation, maintenance, and updating of the Cancer Prevention Study-II (CPS-II) cohort. This study was conducted with Institutional Review Board approval.

CRCGEN: Colorectal Cancer Genetics & Genomics, Spanish study was supported by Instituto de Salud Carlos III, co-funded by FEDER funds –a way to build Europe– (grants PI14-613 and PI09-1286), Agency for Management of University and Research Grants (AGAUR) of the Catalan Government (grant 2017SGR723), and Junta de Castilla y León (grant LE22A10-2). Sample collection of this work was supported by the Xarxa de Bancs de Tumors de Catalunya sponsored by Pla Director d'Oncología de Catalunya (XBTC), Plataforma Biobancos PT13/0010/0013 and ICOBIOBANC, sponsored by the Catalan Institute of Oncology.

Czech Republic CCS: This work was supported by the Grant Agency of the Czech Republic (grants CZ GA CR: GAP304/10/1286 and 1585) and by the Grant Agency of the Ministry of Health of the Czech Republic (grants AZV 15-27580A and AZV 17-30920A).

DACHS: This work was supported by the German Research Council (BR 1704/6-1, BR 1704/6-3, BR 1704/6-4, CH 117/1-1, HO 5117/2-1, HE 5998/2-1, KL 2354/3-1, RO 2270/8-1 and BR 1704/17-1), the Interdisciplinary Research Program of the National Center for Tumor Diseases (NCT), Germany, and the German Federal Ministry of Education and Research (01KH0404, 01ER0814, 01ER0815, 01ER1505A and 01ER1505B).

DALS: National Institutes of Health (R01 CA48998 to M. L. Slattery).

EDRN: This work is funded and supported by the NCI, EDRN Grant (U01 CA 84968-06).

EPIC: The coordination of EPIC is financially supported by International Agency for Research on

Cancer (IARC) and also by the Department of Epidemiology and Biostatistics, School of Public Health, Imperial College London which has additional infrastructure support provided by the NIHR Imperial Biomedical Research Centre (BRC).

The national cohorts are supported by: Danish Cancer Society (Denmark); Ligue Contre le Cancer, Institut Gustave Roussy, Mutuelle Générale de l'Education Nationale, Institut National de la Santé et de la Recherche Médicale (INSERM) (France); German Cancer Aid, German Cancer Research Center (DKFZ), German Institute of Human Nutrition Potsdam- Rehbruecke (DIfE), Federal Ministry of Education and Research (BMBF) (Germany); Associazione Italiana per la Ricerca sul Cancro-AIRC-Italy, Compagnia di SanPaolo and National Research Council (Italy); Dutch Ministry of Public Health, Welfare and Sports (VWS), Netherlands Cancer Registry (NKR), LK Research Funds, Dutch Prevention Funds, Dutch ZON (Zorg Onderzoek Nederland), World Cancer Research Fund (WCRF), Statistics Netherlands (The Netherlands); Health Research Fund (FIS) - Instituto de Salud Carlos III (ISCIII), Regional Governments of Andalucía, Asturias, Basque Country, Murcia and Navarra, and the Catalan Institute of Oncology - ICO (Spain); Swedish Cancer Society, Swedish Research Council and County Councils of Skåne and Västerbotten (Sweden); Cancer Research UK (14136 to EPIC-Norfolk; C8221/A29017 to EPIC-Oxford), Medical Research Council (1000143 to EPIC-Norfolk; MR/M012190/1 to EPIC-Oxford). (United Kingdom).

EPICOLON: This work was supported by grants from Fondo de Investigación Sanitaria/FEDER (PI08/0024, PI08/1276, PS09/02368, P111/00219, PI11/00681, PI14/00173, PI14/00230, PI17/00509, 17/00878, Acción Transversal de Cáncer), Xunta de Galicia (PGIDIT07PXIB9101209PR), Ministerio de Economia y Competitividad (SAF07-64873, SAF 2010-19273, SAF2014-54453R), Fundación Científica de la Asociación Española contra el Cáncer (GCB13131592CAST), Beca Grupo de Trabajo "Oncología" AEG (Asociación Española de Gastroenterología), Fundación Privada Olga Torres, FP7 CHIBCHA Consortium, Agència de Gestió d'Ajuts Universitaris i de Recerca (AGAUR, Generalitat de Catalunya, 2014SGR135, 2014SGR255, 2017SGR21, 2017SGR653), Catalan Tumour Bank Network (Pla Director d'Oncologia, Generalitat de Catalunya), PERIS (SLT002/16/00398, Generalitat de Catalunya), CERCA Programme (Generalitat de Catalunya) and COST Action BM1206 and CA17118. CIBERehd is funded by the Instituto de Salud Carlos III.

ESTHER/VERDI. This work was supported by grants from the Baden-Württemberg Ministry of Science, Research and Arts and the German Cancer Aid.

Harvard cohorts (HPFS, NHS, PHS): HPFS is supported by the National Institutes of Health (P01 CA055075, UM1 CA167552, U01 CA167552, R01 CA137178, R01 CA151993, R35 CA197735, K07 CA190673, and P50 CA127003), NHS by the National Institutes of Health (R01 CA137178, P01 CA087969, UM1 CA186107, R01 CA151993, R35 CA197735, K07CA190673, and P50 CA127003) and PHS by the National Institutes of Health (R01 CA042182).

Hawaii Adenoma Study: NCI grants R01 CA72520.

HCES-CRC: the Hwasun Cancer Epidemiology Study–Colon and Rectum Cancer (HCES-CRC; grants from Chonnam National University Hwasun Hospital, HCRI15011-1).

Kentucky: This work was supported by the following grant support: Clinical Investigator Award from Damon Runyon Cancer Research Foundation (CI-8); NCI R01CA136726.

LCCS: The Leeds Colorectal Cancer Study was funded by the Food Standards Agency and Cancer Research UK Programme Award (C588/A19167).

MCCS cohort recruitment was funded by VicHealth and Cancer Council Victoria. The MCCS was further supported by Australian NHMRC grants 509348, 209057, 251553 and 504711 and by infrastructure provided by Cancer Council Victoria. Cases and their vital status were ascertained through the Victorian Cancer Registry (VCR) and the Australian Institute of Health and Welfare (AIHW), including the National Death Index and the Australian Cancer Database.

MEC: National Institutes of Health (R37 CA54281, P01 CA033619, and R01 CA063464).

MECC: This work was supported by the National Institutes of Health, U.S. Department of Health and Human Services (R01 CA81488 to SBG and GR).

MSKCC: The work at Sloan Kettering in New York was supported by the Robert and Kate Niehaus Center for Inherited Cancer Genomics and the Romeo Milio Foundation. Moffitt: This work was supported by funding from the National Institutes of Health (grant numbers R01 CA189184, P30 CA076292), Florida Department of Health Bankhead-Coley Grant 09BN-13, and the University of South Florida Oehler Foundation. Moffitt contributions were supported in part by the Total Cancer Care Initiative, Collaborative Data Services Core, and Tissue Core at the H. Lee Moffitt Cancer Center & Research Institute, a National Cancer Institute-designated Comprehensive Cancer Center (grant number P30 CA076292).

NCCCS I & II: We acknowledge funding support for this project from the National Institutes of Health, R01 CA66635 and P30 DK034987.

NFCCR: This work was supported by an Interdisciplinary Health Research Team award from the Canadian Institutes of Health Research (CRT 43821); the National Institutes of Health, U.S. Department of Health and Human Serivces (U01 CA74783); and National Cancer Institute of Canada grants (18223 and 18226). The authors wish to acknowledge the contribution of Alexandre Belisle and the genotyping team of the McGill University and Génome Québec Innovation Centre, Montréal, Canada, for genotyping the Sequenom panel in the NFCCR samples. Funding was provided to Michael O. Woods by the Canadian Cancer Society Research Institute.

NSHDS: Swedish Research Council; Swedish Cancer Society; Cutting-Edge Research Grant and other grants from Region Västerbotten; Knut and Alice Wallenberg Foundation; Lion's Cancer Research Foundation at Umeå University; the Cancer Research Foundation in Northern Sweden; and the Faculty of Medicine, Umeå University, Umeå, Sweden.

OFCCR: The Ontario Familial Colorectal Cancer Registry was supported in part by the National Cancer Institute (NCI) of the National Institutes of Health (NIH) under award U01 CA167551 and award U01/U24 CA074783 (to SG). Additional funding for the OFCCR and ARCTIC testing and genetic analysis was through and a Canadian Cancer Society CaRE (Cancer Risk Evaluation) program grant and Ontario Research Fund award GL201-043 (to BWZ), through the Canadian Institutes of Health Research award 112746 (to TJH), and through generous support from the Ontario Ministry of Research and Innovation.

OSUMC: OCCPI funding was provided by Pelotonia and HNPCC funding was provided by the NCI (CA16058 and CA67941).

PLCO: Intramural Research Program of the Division of Cancer Epidemiology and Genetics and supported by contracts from the Division of Cancer Prevention, National Cancer Institute, NIH, DHHS. Funding was provided by National Institutes of Health (NIH), Genes, Environment and Health Initiative (GEI) Z01 CP 010200, NIH U01 HG004446, and NIH GEI U01 HG 004438.

SCCFR: The Seattle Colon Cancer Family Registry was supported in part by the National Cancer Institute (NCI) of the National Institutes of Health (NIH) under awards U01 CA167551, U01 CA074794 (to JDP), and awards U24 CA074794 and R01 CA076366 (to PAN).

SEARCH: The University of Cambridge has received salary support in respect of PDPP from the NHS in the East of England through the Clinical Academic Reserve. Cancer Research UK (C490/A16561); the UK National Institute for Health Research Biomedical Research Centres at the University of Cambridge.

SELECT: Research reported in this publication was supported in part by the National Cancer Institute of the National Institutes of Health under Award Numbers U10 CA37429 (CD Blanke), and UM1 CA182883 (CM Tangen/IM Thompson). The content is solely the responsibility of the authors and does not necessarily represent the official views of the National Institutes of Health.

SMS and REACH: This work was supported by the National Cancer Institute (grant P01 CA074184 to J.D.P. and P.A.N., grants R01 CA097325, R03 CA153323, and K05 CA152715 to P.A.N., and the National Center for Advancing Translational Sciences at the National Institutes of Health (grant KL2 TR000421 to A.N.B.-H.)

The Swedish Low-risk Colorectal Cancer Study: The study was supported by grants from the Swedish research council; K2015-55X-22674-01-4, K2008-55X-20157-03-3, K2006-72X-20157-01-2 and the Stockholm County Council (ALF project).

Swedish Mammography Cohort and Cohort of Swedish Men: This work is supported by the Swedish Research Council /Infrastructure grant, the Swedish Cancer Foundation, and the Karolinska Institute´s Distinguished Professor Award to Alicja Wolk.

UK Biobank: This research has been conducted using the UK Biobank Resource under Application Number 8614

VITAL: National Institutes of Health (K05 CA154337).

WHI: The WHI program is funded by the National Heart, Lung, and Blood Institute, National Institutes of Health, U.S. Department of Health and Human Services through contracts HHSN268201100046C, HHSN268201100001C, HHSN268201100002C, HHSN268201100003C, HHSN268201100004C, and HHSN271201100004C.

Acknowledgements:

ASTERISK: We are very grateful to Dr. Bruno Buecher without whom this project would not have existed. We also thank all those who agreed to participate in this study, including the patients and the healthy control persons, as well as all the physicians, technicians and students.

CLUE: We appreciate the continued efforts of the staff members at the Johns Hopkins George W. Comstock Center for Public Health Research and Prevention in the conduct of the CLUE II study. We thank the participants in CLUE. Cancer incidence data for CLUE were provided by the Maryland Cancer

Registry, Center for Cancer Surveillance and Control, Maryland Department of Health, 201 W. Preston Street, Room 400, Baltimore, MD 21201, http://phpa.dhmh.maryland.gov/cancer, 410-767-4055. We acknowledge the State of Maryland, the Maryland Cigarette Restitution Fund, and the National Program of Cancer Registries of the Centers for Disease Control and Prevention for the funds that support the collection and availability of the cancer registry data.

COLON and NQplus: the authors would like to thank the COLON and NQplus investigators at Wageningen University & Research and the involved clinicians in the participating hospitals.

CORSA: We kindly thank all those who contributed to the screening project Burgenland against CRC. Furthermore, we are grateful to Doris Mejri and Monika Hunjadi for laboratory assistance.

CPS-II: The authors thank the CPS-II participants and Study Management Group for their invaluable contributions to this research. The authors would also like to acknowledge the contribution to this study from central cancer registries supported through the Centers for Disease Control and Prevention National Program of Cancer Registries, and cancer registries supported by the National Cancer Institute Surveillance Epidemiology and End Results program.

Czech Republic CCS: We are thankful to all clinicians in major hospitals in the Czech Republic, without whom the study would not be practicable. We are also sincerely grateful to all patients participating in this study.

DACHS: We thank all participants and cooperating clinicians, and Ute Handte-Daub, Utz Benscheid, Muhabbet Celik and Ursula Eilber for excellent technical assistance.

EDRN: We acknowledge all the following contributors to the development of the resource: University of Pittsburgh School of Medicine, Department of Gastroenterology, Hepatology and Nutrition: Lynda Dzubinski; University of Pittsburgh School of Medicine, Department of Pathology: Michelle Bisceglia; and University of Pittsburgh School of Medicine, Department of Biomedical Informatics.

EPIC: Where authors are identified as personnel of the International Agency for Research on Cancer/World Health Organization, the authors alone are responsible for the views expressed in this article and they do not necessarily represent the decisions, policy or views of the International Agency for Research on Cancer/World Health Organization.

EPICOLON: We are sincerely grateful to all patients participating in this study who were recruited as part of the EPICOLON project. We acknowledge the Spanish National DNA Bank, Biobank of Hospital Clínic–IDIBAPS and Biobanco Vasco for the availability of the samples. The work was carried out (in part) at the Esther Koplowitz Centre, Barcelona.

Harvard cohorts (HPFS, NHS, PHS): The study protocol was approved by the institutional review boards of the Brigham and Women’s Hospital and Harvard T.H. Chan School of Public Health, and those of participating registries as required. We acknowledge Channing Division of Network Medicine, Department of Medicine, Brigham and Women's Hospital as home of the NHS. We would like to thank the participants and staff of the HPFS, NHS and PHS for their valuable contributions as well as the following state cancer registries for their help: AL, AZ, AR, CA, CO, CT, DE, FL, GA, ID, IL, IN, IA, KY, LA, ME, MD, MA, MI, NE, NH, NJ, NY, NC, ND, OH, OK, OR, PA, RI, SC, TN, TX, VA, WA, WY. The authors assume full responsibility for analyses and interpretation of these data.

Kentucky: We would like to acknowledge the staff at the Kentucky Cancer Registry.

LCCS: We acknowledge the contributions of Jennifer Barrett, Robin Waxman, Gillian Smith and Emma Northwood in conducting this study.

NCCCS I & II: We would like to thank the study participants, and the NC Colorectal Cancer Study staff.

NSHDS investigators thank the Biobank Research Unit at Umeå University, the Västerbotten Intervention Programme, the Northern Sweden MONICA study and Region Västerbotten for providing data and samples and acknowledge the contribution from Biobank Sweden, supported by the Swedish Research Council (VR 2017-00650).

PLCO: The authors thank the PLCO Cancer Screening Trial screening center investigators and the staff from Information Management Services Inc and Westat Inc. Most importantly, we thank the study participants for their contributions that made this study possible.

SCCFR: The authors would like to thank the study participants and staff of the Seattle Colon Cancer Family Registry and the Hormones and Colon Cancer study (CORE Studies).

SEARCH: We thank the SEARCH team.

SELECT: We thank the research and clinical staff at the sites that participated on SELECT study, without whom the trial would not have been successful. We are also grateful to the 35,533 dedicated men who participated in SELECT.

WHI: The authors thank the WHI investigators and staff for their dedication, and the study participants for making the program possible. A full listing of WHI investigators can be found at: http://www.whi.org/researchers/Documents%20%20Write%20a%20Paper/WHI%20Investigator%20Short%20List.pdf
